# Lipid A Modification and Metabolic Adaptation in Polymyxin-Resistant, New Delhi Metallo-β-Lactamase–Producing Klebsiella pneumoniae

**DOI:** 10.1128/spectrum.00852-23

**Published:** 2023-07-11

**Authors:** Jing Lu, Meiling Han, Heidi H. Yu, Phillip J. Bergen, Yiyun Liu, Jinxin Zhao, Hasini Wickremasinghe, Xukai Jiang, Yang Hu, Haiyan Du, Yan Zhu, Tony Velkov

**Affiliations:** a Infection Program, Department of Microbiology, Biomedicine Discovery Institute, Monash University, Melbourne, Victoria, Australia; b National Risk Assessment Laboratory for Antimicrobial Resistance of Animal Original Bacteria, College of Veterinary Medicine, South China Agricultural University, Guangzhou, Guangdong, China; c Department of Pharmacology, The Faculty of Medicine Nursing and Health Sciences, Monash University, Melbourne, Victoria, Australia; Texas A&M University

**Keywords:** *Klebsiella pneumoniae*, polymyxin, lipid A modification, metabolomics, lipidomics, *phoQ*

## Abstract

Polymyxins are last-line antibiotics employed against multidrug-resistant (MDR) Klebsiella pneumoniae. Worryingly, polymyxin resistance is rapidly on the rise globally. Polymyxins initially target lipid A of lipopolysaccharides (LPSs) in the cell outer membrane (OM), causing disorganization and cell lysis. While most studies focus on how genetic variations confer polymyxin resistance, the mechanisms of membrane remodeling and metabolic changes in polymyxin-resistant strains remain unclear, thus hampering the development of effective therapies to treat severe K. pneumoniae infections. In the present study, lipid A profiling, OM lipidomics, genomics, and metabolomics were integrated to elucidate the global mechanisms of polymyxin resistance and metabolic adaptation in a polymyxin-resistant strain (strain S01R; MIC of >128 mg/L) obtained from K. pneumoniae strain S01, a polymyxin-susceptible (MIC of 2 mg/L), New Delhi metallo-β-lactamase (NDM)-producing MDR clinical isolate. Genomic analysis revealed a novel in-frame deletion at position V258 of PhoQ in S01R, potentially leading to lipid A modification with 4-amino-4-deoxy-l-arabinose (L-Ara4N) despite the absence of polymyxin B. Comparative metabolomic analysis revealed slightly elevated levels of energy production and amino acid metabolism in S01R compared to their levels in S01. Exposure to polymyxin B (4 mg/L for S01 and 512 mg/L for S01R) substantially altered energy, nucleotide, and amino acid metabolism and resulted in greater accumulation of lipids in both strains. Furthermore, the change induced by polymyxin B treatment was dramatic at both 1 and 4 h in S01 but only significant at 4 h in S01R. Overall, profound metabolic adaptation was observed in S01R following polymyxin B treatment. These findings contribute to our understanding of polymyxin resistance mechanisms in problematic NDM-producing K. pneumoniae strains and may facilitate the discovery of novel therapeutic targets.

**IMPORTANCE** Antimicrobial resistance (AMR) is a major threat to global health. The emergence of resistance to the polymyxins that are the last line of defense in so-called Gram-negative “superbugs” has further increased the urgency to develop novel therapies. There are frequent outbreaks of K. pneumoniae infections in hospitals being reported, and polymyxin usage is increasing remarkably. Importantly, the polymyxin-resistant K. pneumoniae strains are imposing more severe consequences to health systems. Using metabolomics, lipid A profiling, and outer membrane lipidomics, our findings reveal (i) changes in the pentose phosphate pathway and amino acid and nucleotide metabolism in a susceptible strain following polymyxin treatment and (ii) how cellular metabolism, lipid A modification, and outer membrane remodeling were altered in K. pneumoniae following the acquisition of polymyxin resistance. Our study provides, for the first time, mechanistic insights into metabolic responses to polymyxin treatment in a multidrug-resistant, NDM-producing K. pneumoniae clinical isolate with acquired polymyxin resistance. Overall, these results will assist in identifying new therapeutic targets to combat and prevent polymyxin resistance.

## INTRODUCTION

Multidrug-resistant (MDR) Klebsiella pneumoniae is responsible for a plethora of nosocomial infections, including ventilator-associated pneumonia, urinary tract infection, sepsis, wound and catheter-related infections, and bacterial meningitis (1–3). Polymyxins (polymyxin B and colistin) are a group of cationic lipopeptide antibiotics that are often utilized as a last-line treatment for problematic MDR, Gram-negative bacterial infections (4). While the exact mechanism(s) by which polymyxins exert their antibacterial activity remains unclear, what is known is that the mechanism involves initial electrostatic interactions between positively charged α,γ-diaminobutyric acid (Dab) residues of polymyxin molecules and negatively-charged phosphate groups of lipid A in the Gram-negative bacterial outer membrane (OM) (5). Following this initial OM binding event, the fatty acyl tail of polymyxin anchors into the hydrophobic region of the OM lipid bilayer, causing membrane disorganization and eventual cell death (6).

Of great concern is that the increased use and misuse of polymyxins globally (7, 8) has eventuated in wide-spread polymyxin-resistant pathogens in both the community and nosocomial settings (2, 9, 10). In K. pneumoniae, resistance is mediated by lipid A modifications with positively charged 4-amino-4-deoxy-l-arabinose (L-Ara4N; mediated by *arnBCADTEF*) (11) and/or phosphoethanolamine (pEtN; mediated by *pmrC* or *mcr*) (12, 13), which repel the like-charged polymyxin and thereby attenuate the initial electrostatic interactions of the polymyxin molecules with the bacterial OM (14). Upon polymyxin exposure, the membrane sensor kinases PhoQ and/or PmrB activate their respective cognate response regulators PhoP and/or PmrA, which in turn induce the expression of *arn* genes or *pmrC* (a chromosomal gene encoding pEtN transferase) (15). Mutations in *phoQ* and *pmrB* may cause constitutive activation of their respective cognate response regulators regardless of polymyxins, with subsequent increased expression of lipid A modification genes, thereby conferring polymyxin resistance (11, 16–19). Loss-of-function mutations in *mgrB*, which encodes the membrane protein MgrB that inhibits the kinase activity of PhoQ, also result in polymyxin resistance (18).

In the present study, we employed K. pneumoniae strain S01, an MDR clinical isolate harboring an IncN2-type *bla*_NDM-1_-carrying plasmid that is resistant to a variety of antimicrobials but susceptible to polymyxins and amikacin (20). We previously employed transcriptomics and metabolomics to investigate the cellular responses of S01 to polymyxin monotherapy and combination therapy with chloramphenicol (21, 22). However, it remains unclear how bacterial metabolism in K. pneumoniae is rewired after the acquisition of polymyxin resistance. Therefore, in the present report, we combined lipid A profiling, OM lipidomics, genomics, and metabolomics to investigate the membrane remodeling and metabolic changes of a polymyxin-resistant mutant (strain S01R) derived from S01 through serial passaging in the presence of polymyxin B. The results presented herein serve to significantly progress our mechanistic understanding of polymyxin resistance and provide key insights for optimization of polymyxin treatments against this problematic Gram-negative pathogen.

## RESULTS

### Lipid A modifications and altered outer membrane glycerophospholipids in strain S01R.

Genomic analysis revealed that S01R contains a never-before-reported 3-nucleotide (nt) in-frame deletion in sensor kinase gene *phoQ*, leading to the deletion of valine at position 258 in the protein PhoQ (Val258del). PhoQ is the major component of the key two-component system (TCS) PhoPQ associated with bacterial virulence and polymyxin resistance in K. pneumoniae (23). In addition, a 7-day *in vitro* passaging study showed that resistance to polymyxin B was stable over the passaging period for S01R (Table S1 in the supplemental material), indicating a low potential for loss of resistance in this mutant.

**FIG 1:**
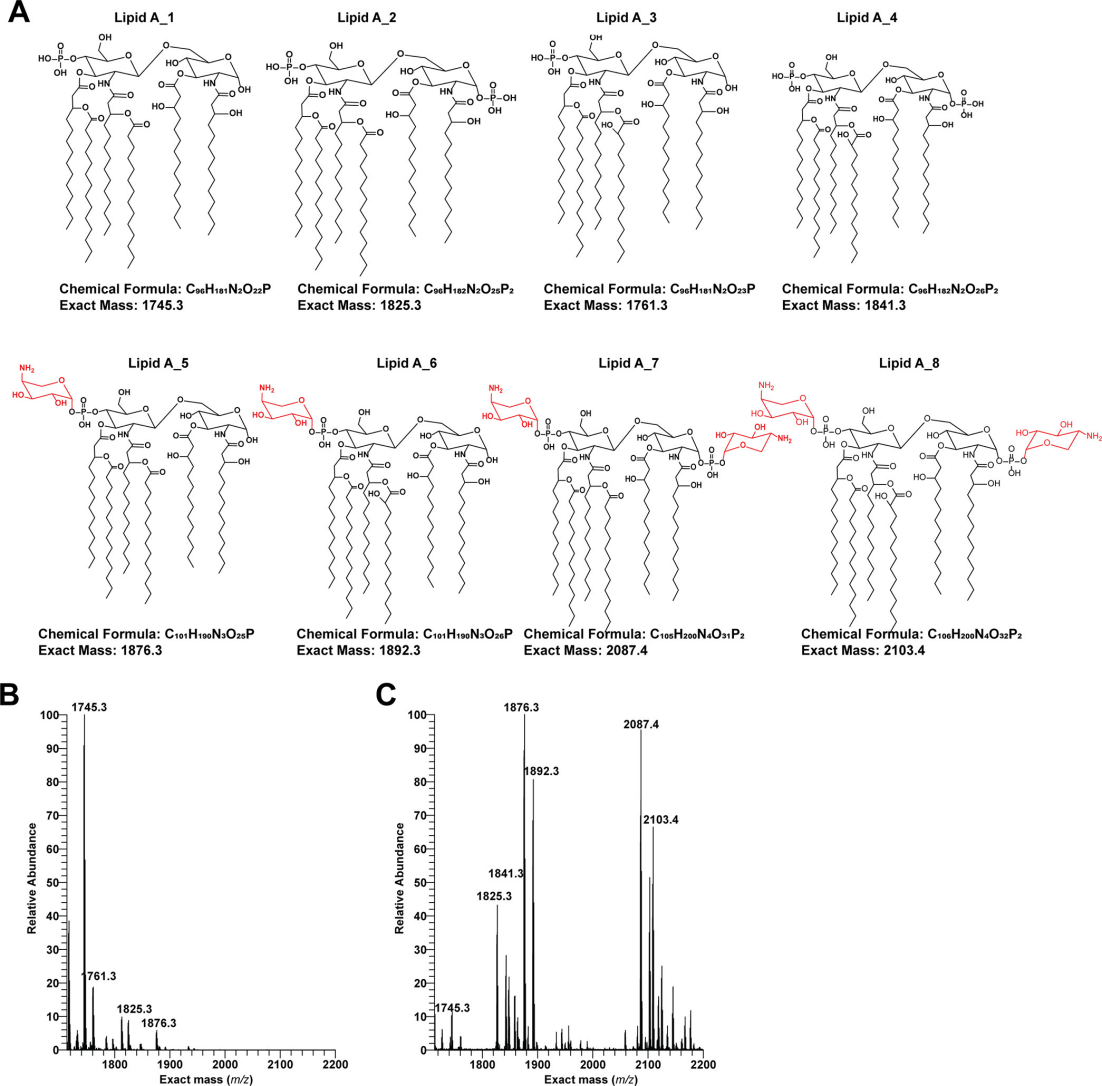
Lipid A profiling of K. pneumoniae strains S01 and S01R in the absence of polymyxin B. (A) Lipid A structures from S01 and S01R. (B, C) Relative abundance and *m/z* values of lipid A detected in S01 (B) and S01R (C) in the absence of polymyxin treatment. Relative abundance and *m/z* values were determined by LC-MS. The L-Ara4N is shown in red color.

Lipid A profiling shows that, in the absence of polymyxin B, strain S01 contains two major lipid A species, namely, hexa-acylated (C_14_) structures consisting of either a monophosphate (*m/z* 1,745.3) or biphosphate (*m/z* 1,825.3) (Fig. 1A). A not-so-common hydroxylation (-OH) on the C′-2 fatty acyl chain fatty acids (*m/z* 1,761.3) was the second most abundant species observed. Surprisingly, lipid A modifications with only one L-Ara4N at the C-1′ or C-4′ position (*m/z* 1,876.3) were also detected in S01, but with low abundance (Fig. 1B). In S01R, one (*m/z* 1,876.3 and 1,892.3) or two (*m/z* 2,087.4 and 2,109.4) L-Ara4N groups were present on lipid A, which were also the most abundant lipid A species (Fig. 1C), indicating a very active L-Ara4N synthesis pathway.

Overall, with OM lipidomics, 89 and 91 glycerophospholipid (GPL) species were identified in the OM in S01 and S01R, respectively. Compared to the wild-type S01, the mutant S01R contained significantly enhanced levels of particular GPL species, including phosphatidylethanolamine (PE; 31:2, 32:2, 33:2, 35:2, 35:3, and 36:1), lysophosphatidylethanolamine (lysoPE; 16:1, 18:1, and 18:2), cardiolipin (CL; 68:2 and 74:3), and phosphatidylcholine (PC; 35:2), but significantly decreased levels of phosphatidylglycerol (PG; 30:0 and 32:1) (Fig. S1). It is tempting to speculate that the altered GPL composition in the OM may result from changes in membrane polarity and/or rigidity due to lipid A modifications.

### Global comparative metabolomics analysis of S01 versus S01R.

**FIG 2:**
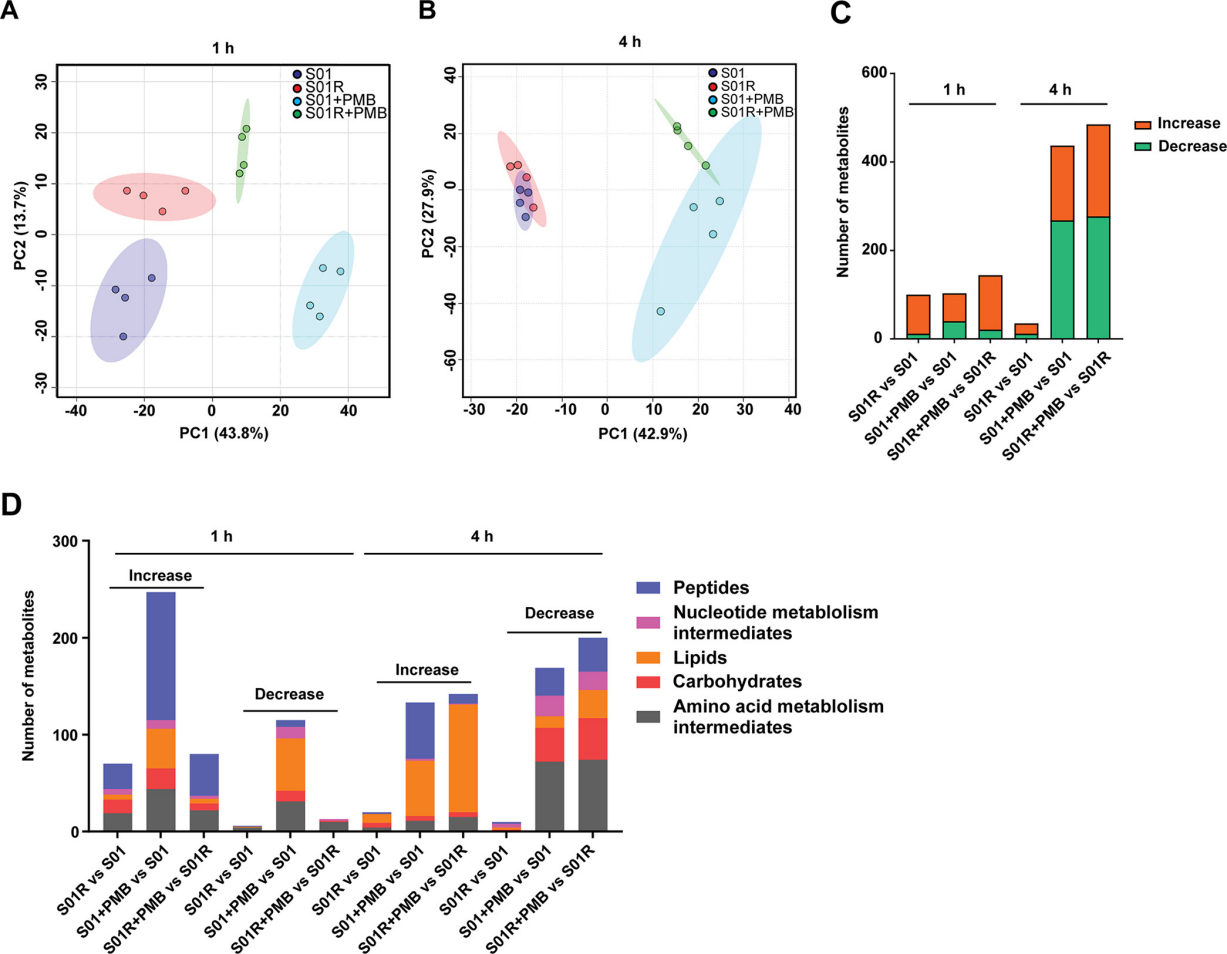
Total changed metabolites in strains S01 and S01R. (A, B) Principal-component analysis (PCA) plots showing the distribution of metabolites in S01 and S01R with or without polymyxin B treatment at 1 h (A) and 4 h (B). (C) Numbers of significantly increased and decreased metabolites overall at 1 h and 4 h after treatment with polymyxin B. (D) Numbers of significantly changed metabolite types or involved pathways.

The fitness of strain S01R was not significantly influenced compared to that of strain S01 in the growth curve (Fig. S2). Time-kill studies were performed to observe the polymyxin killing effect on strains S01 and S01R exposed to a range of polymyxin concentrations selected based on their MICs (Fig. S2). To further explore the polymyxin resistance mechanism(s) in S01R, we conducted an untargeted comparative metabolomics analysis of S01 versus S01R and examined their responses following polymyxin B exposure. Principal-component analysis (PCA) revealed dramatic heterogeneity among all groups at 1 h (Fig. 2A). At this time, there were substantial metabolomic differences between S01R and S01 in both the absence and presence of polymyxin B, with both strains exhibiting different metabolic patterns that indicated metabolic adaptations in intrinsic (i.e., S01R) and acquired (i.e., S01) polymyxin resistance (Fig. 2A). Although at 4 h, the metabolite differences between S01 and S01R were minimal in the absence of polymyxin B, polymyxin B exposure resulted in substantial changes in the metabolomes of both strains compared to those of the respective untreated controls (Fig. 2B). In the absence of polymyxin B, the levels of 100 and 35 metabolites were significantly changed in S01R compared to their levels in S01 at 1 and 4 h, respectively (fold change of ≥2, false-discovery rate [FDR] of <0.05) (Fig. 2C). Generally, compared to S01, S01R displayed significant perturbations across peptide, carbohydrate, amino acid, nucleotide, and lipid metabolism that were coincident with the acquisition of the aforementioned resistance-conferring mutation in *phoQ* (Fig. 2D), suggesting that activation of PhoPQ impacted cellular metabolism.

Notably, the polymyxin-induced metabolic changes became more dramatic for S01R at 4 h than they were at 1 h. In contrast, the metabolic changes in S01 were pronounced from the earlier sampling time (1 h) posttreatment. Following polymyxin B treatment, 362 (247 increased and 115 decreased) and 437 (169 increased and 268 decreased) metabolites in S01 were significantly changed at 1 and 4 h, respectively; the corresponding numbers in S01R were 101 (80 increased and 21 decreased) and 342 (142 increased and 200 decreased) (Fig. 2C). In both strains, a large number of peptide and lipid metabolites were significantly increased, while intermediates of amino acid metabolism were depleted, in particular at 4 h post-polymyxin B exposure. This is possibly reflective of an increased demand for lipids that feed into the membrane remodeling processes in response to polymyxin treatment (Fig. 2D).

### Significant metabolic changes in S01R compared to S01 in the absence of polymyxin B.

**FIG 3:**
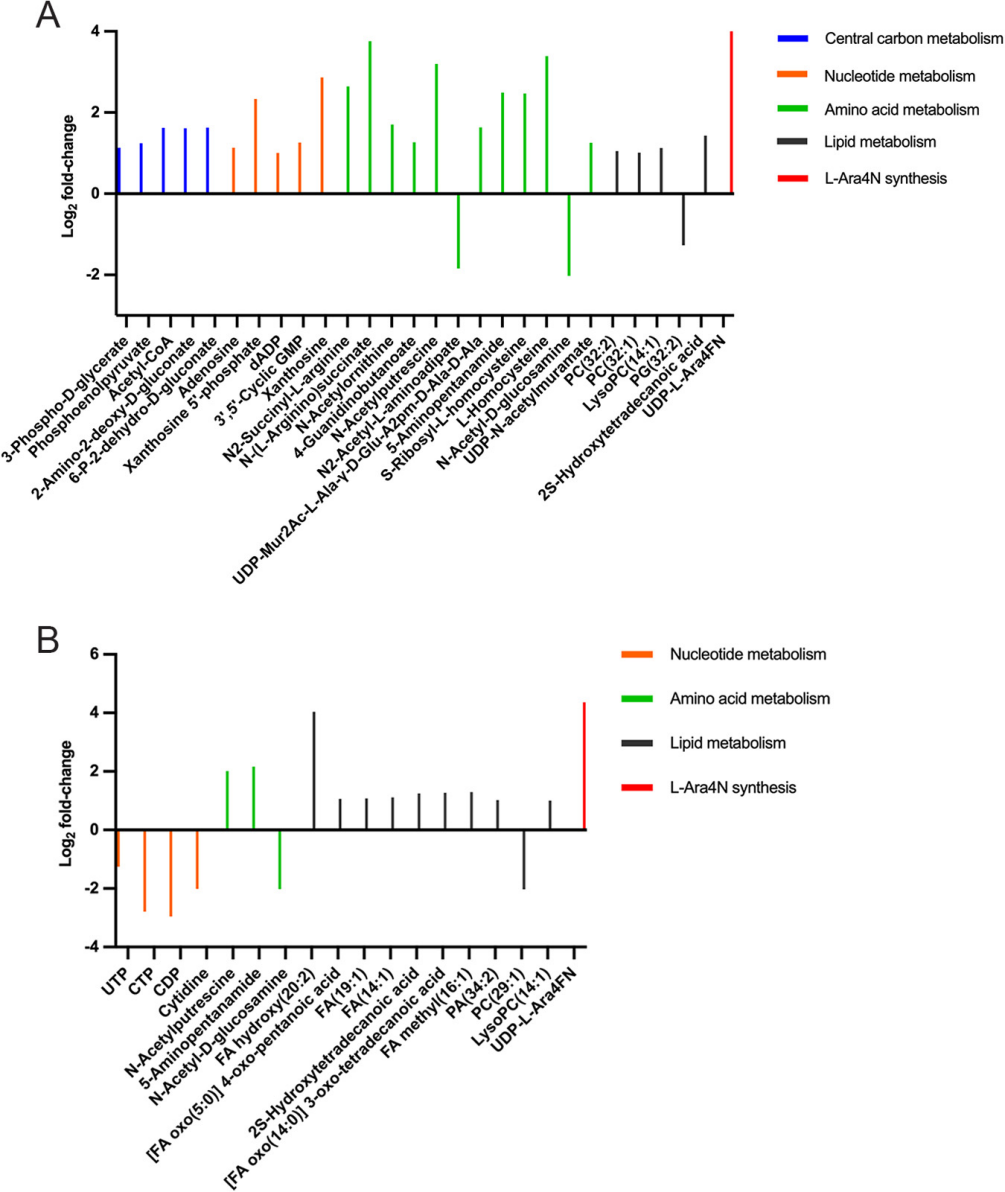
Significantly changed metabolites in S01R compared with their levels in S01. (A) Metabolites that were significantly changed in strain S01R at 1 h and their types or the metabolism pathways involved. (B) Metabolites that were significantly changed in S01R at 4 h and their types or the metabolism pathways involved.

In strain S01R, these significant changed metabolites were involved in central carbon, purine, lipids and amino acid metabolism (Fig. 3A). There were a small number of pyrimidine metabolites that were decreased but a greater increase of lipid metabolites at 4 h (Fig. 3B). At 1 h, *N*-acetyl-d-glucosamine, the common precursor of peptidoglycan and lipid A biosynthesis, was decreased by 4.1-fold, while UDP-*N*-acetylmuramate, which is also involved in peptidoglycan biosynthesis and additionally feeds into phosphatidylcholine biosynthesis, was increased by 2.4-fold (Fig. 3A) (24). At 4 h, several intermediates associated with fatty acid metabolism were increased in S01R, suggesting a significant change of cellular lipid components following lipid A modification. Surprisingly, a number of key pyrimidine metabolites, including cytidine, CDP, CTP, and UTP, were significantly decreased at this time point. Notably, 8 metabolites were commonly increased at both 1 and 4 h in S01R, including *N*-acetylputrescine from arginine metabolism (9.2- and 4.1-fold increases at 1 and 4 h, respectively) and 5-aminopentanamide from lysine metabolism (5.6- and 4.5-fold increases), suggesting that arginine and lysine might play a role in the resistance in S01R. As expected, enormous 81- and 20-fold increases of UDP-β-(4-deoxy-4-formamido-l-arabinose) (UDP-L-Ara4FN) were observed in S01R compared to their levels in S01 at 1 and 4 h, respectively, suggesting a highly active L-Ara4N biosynthesis flux toward lipid A modification (i.e., UDP-L-Ara4FN is the precursor of L-Ara4N) (Fig. 3A and B) (18). Collectively, these results revealed that the metabolism of S01R was distinct from that of its wild-type parent strain S01, especially in relation to the markedly increased levels of L-Ara4N precursors that feed into the lipid A modification pathway induced by the TCS response.

### Metabolic changes in response to polymyxin B treatment in S01 and S01R. (i) Perturbations to central carbon metabolism.

**FIG 4:**
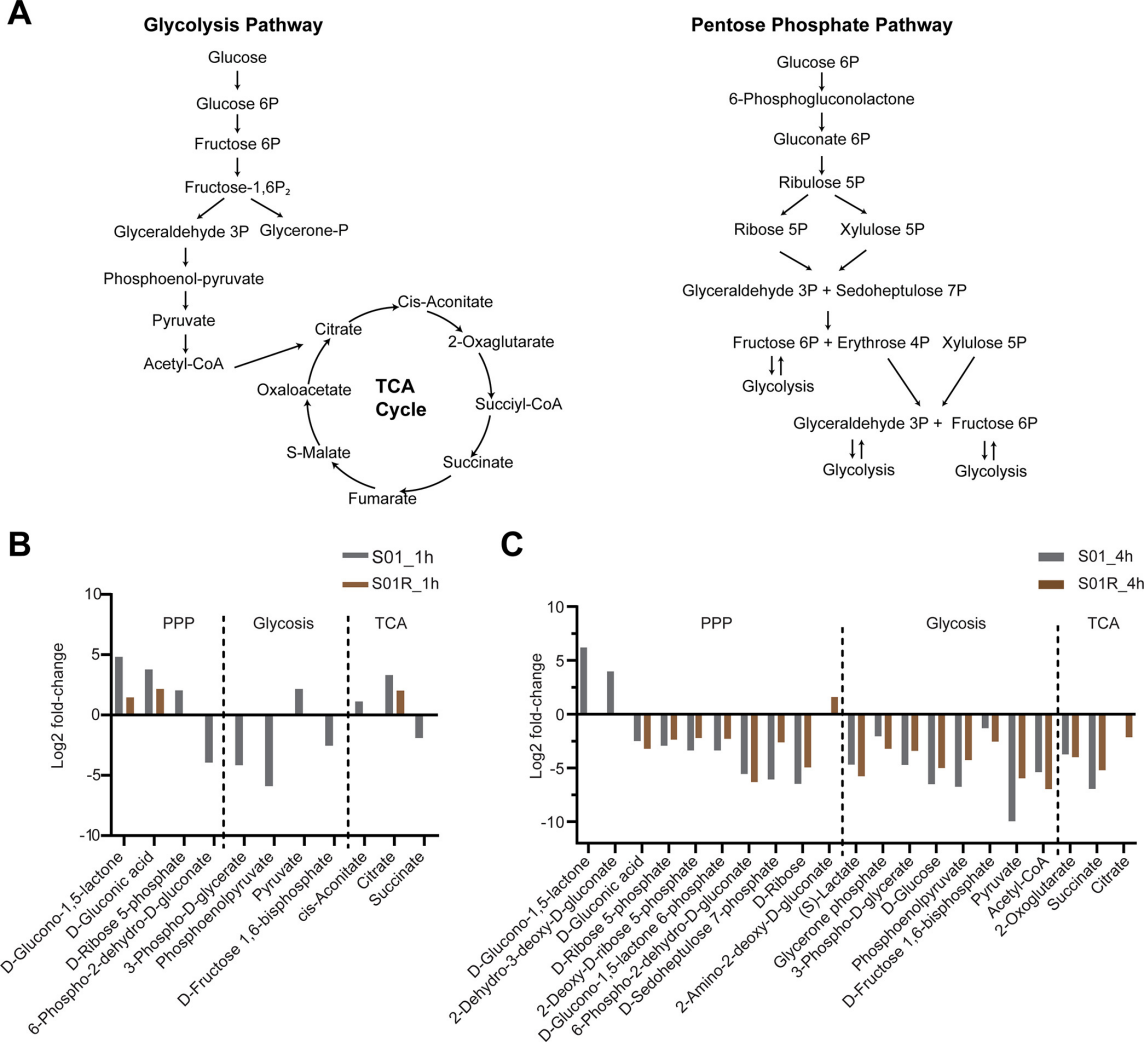
Significantly changed metabolites associated with central carbon metabolism following polymyxin B treatment. (A) Diagram of the central carbon pathway, including glycolysis, the tricarboxylic acid (TCA) cycle, and the pentose phosphate pathway (PPP). (B, C) Log_2_ fold changes of the significantly changed metabolites associated with central carbon metabolism following 1 h (B) and 4 h (C) of polymyxin B exposure.

Treatment with polymyxin B for 1 h significantly altered the central carbon metabolism of S01; this was reflected by the increased metabolites in the pentose phosphate pathway (PPP) and perturbations in glycolysis and the tricarboxylic acid (TCA) cycle (Fig. 4). Notably, the metabolome of S01R remained mostly unperturbed following polymyxin B exposure for 1 h, except for moderate increases in d-gluconic acid (4.5-fold), d-glucono-1,5-lactone (2.8-fold), and citrate (4.1-fold) (Fig. 4B). This may result from the fact that the *phoQ* mutation in S01R potentially results in the constitutive modification of lipid A with L-Ara4N, which in turn renders the OM impervious to polymyxin B (Fig. 1).

Profound metabolic perturbations of central carbon metabolism occurred in both S01 and S01R following 4 h of exposure to the respective killing concentrations of polymyxin B (4 mg/L for S01 and 512 mg/L for S01R). Substantial increases of d-glucono-1,5-lactone (73-fold) and d-gluconic acid (15-fold) were observed in the oxidative branch of PPP in S01, suggesting inhibition of the metabolic activity and the accumulation of intermediates, whereas the levels of metabolites of the nonoxidative branch were decreased by 5.6- to 88-fold in S01 and 5.5- to 79-fold in S01R. Metabolites from glycolysis were also decreased in both S01 (2.5- to 994-fold) and S01R (5.9- to 125-fold). Notably, acetyl-coenzyme A (CoA) was decreased by 994-fold in S01 and 125-fold in S01R, reflecting a state of energy depletion, as acetyl-CoA is an essential element for ATP production. Likewise, significant depletion of TCA metabolites was observed in both S01 and S01R (Fig. 4C). Collectively, these results showed substantial perturbations of central metabolism in both S01 and S01R due to exposure to the respective killing concentrations of polymyxin B. Although modifications of lipid A with L-Ara4N in S01R protected cells from the initial interaction with polymyxins and cell metabolism thus remained largely unperturbed at 1 h, the protection did not last to 4 h.

### (ii) Upregulation of lipids.

**FIG 5:**
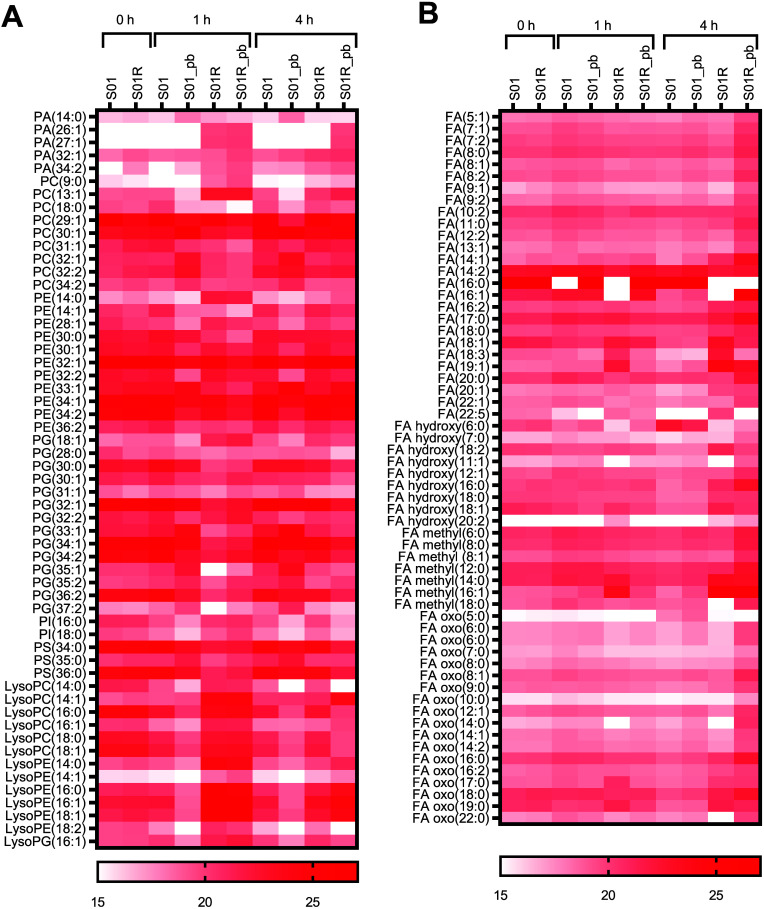
Heat maps showing glycerophospholipid (A) and fatty acid (B) changes following polymyxin B treatment. The relative intensities of the metabolites were log_2_ transformed. pb, polymyxin B treated.

Following 1 h of polymyxin B exposure, 48 and 12 phospholipids were significantly changed in S01 and S01R, respectively (Fig. 5A). In S01, there were significant decreases in both PG species (28:0, 30:0, 30:1, 32:1, 33:1, 34:1, 34:2, 35:1, 35:2, 36:2, and 37:2; range, 2.7- to 31-fold) and PE species [30:0, 32:1, 33:1, 34:2, and 36:2; range, 2.4- to 8.6-fold] but increases in lysoPC, lysoPE, and phosphatidic acid (PA) species (range, 2.1- to 1,148-fold) (Fig. 5A and Data Set S1). Polymyxin B exposure for 1 h in S01R led to decreases in PG (31:1, 33:1, and 35:2; range, 2.1- to 3.1-fold) and increases in lysoPE (range, 2.0- to 32-fold) species, and no significant change in PE species was detected.

The changes in phospholipids following 4 h of treatment were dramatic in both strains, with 44 significant changes observed in S01 and 41 in S01R (Fig. 5A and Data Set S1). In S01, lysoPC, lysoPE, and PA species increased well above the levels observed following 1 h of treatment (range, 2.4- to 1,199-fold), while some PC species (29:1, 30:1, 31:1, 32:1, 32:2, 34:1, and 34:2) decreased (2.6- to 10-fold) and other PC species (9:0 and O-13:1) increased (112- and 203-fold, respectively). Several species of PE (14:0; 28:1; 32:2) increased, whereas others decreased (32:1; 30:0; 33:1). Interestingly, two phosphatidylinositol (PI) species (16:0 and 18:0) were increased in S01 but not in S01R. Most phospholipids were also increased in S01R after exposure to the higher concentration of polymyxin B for 4 h, while some PC species (30:1, 32:1, and 34:2; 4.6-, 2.7- and 4.7-fold, respectively) were decreased and others (29:1, 9:0, and O-13:1; 5.2-, 52.3- and 65.0-fold, respectively) were increased. Eight species of PE (14:0, 28:1, 32:2, 30:1, 32:1, 34:1, and 34:2) were increased in S01R. In addition, 12 species of PG were decreased in S01R, whereas in S01, 6 were decreased but 1 species of PG was increased. Overall, exposure to the killing concentrations of polymyxin B resulted in increases of PE species and decreases of PG species in strains S01 and S01R.

In the case of fatty acids, 1 h of polymyxin B exposure resulted in 21 and 3 significantly changed fatty acids in S01 and S01R, respectively (Fig. 5B and Data Set S2), while by 4 h, the number of significantly changed fatty acids had increased substantially in both strains. In S01, six fatty acids were decreased [oxo(5:0, 14:0, and 22:0), hydroxy(6:0 and 11:1), and methyl(18:0); range, 3.1- to 108-fold] and 24 were increased (oxo, hydroxy, and methyl; range, 2.1- to 990-fold). In S01R, there were significant decreases in oxo (5:0; 6.6-fold) and hydroxy (6:0; 50-fold) fatty acids, as well as significant increases in another 52 fatty acids (range, 2.2- to 213-fold) (Fig. 5B and Data Set S2). These results showed dramatic changes of phospholipids in response to polymyxin B treatment, with particularly large increases of PE species and fatty acids in S01R.

### (iii) Perturbations of amino acid metabolism.

Polymyxin B treatment led to significant perturbations in amino acid metabolism in S01 at both 1 and 4 h. The intermediates mainly affected were from the arginine and lysine pathways. At 1 h, there were 8 increased metabolites and 5 decreased metabolites from the arginine pathway. However, at 4 h, there were significant depletions of arginine metabolites (18 were decreased by 7.1- to 203-fold) (Fig. S3). Likewise, there were 5 increased metabolites and 6 decreased metabolites from the lysine pathway at 1 h, whereas 15 metabolites were decreased at 4 h, with the decreases ranging from 2.74- to 126-fold (Fig. S4).

In S01R, the significant perturbations in amino acid metabolism mainly occurred at 4 h. For arginine metabolism, there were decreases of 4 intermediates (range, 2.0- to 2.8-fold) and 1 increased metabolite (8.0-fold) at 1 h, while at 4 h, 11 metabolites (range, 2.9- to 495-fold) were decreased (Fig. S3). For lysine metabolism in S01R, 3 metabolites (0.3- to 5.7-fold) were changed at 1 h, but at 4 h, 16 were decreased by 5.0- to 406-fold and 2 were increased (Fig. S4).

### (iv) Perturbations of nucleotide metabolism.

In S01, there were significant decreases (2.1- to 35-fold) of metabolites involved with purine metabolism, including nucleosides (guanosine, deoxyadenosine, and xanthosine) and nucleotides (AMP, dADP, dADP, GDP, and GTP), following 1 h of polymyxin B exposure (Fig. S5). However, increases in adenine (9.0-fold) and 3′,5′-cyclic GMP (5.2-fold) were observed (Fig. S5). In S01R, there were 3 increased metabolites involved in pyrimidine metabolism following 1 h of polymyxin B treatment, but 13 metabolites were decreased for both purine and pyrimidine metabolism at 4 h (Fig. S5).

## DISCUSSION

Infections caused by multidrug resistant (MDR), New Delhi metallo-β-lactamase (NDM)-producing K. pneumoniae strains impose a significant health burden on society, and their treatment presents a major challenge for clinicians (25). As polymyxins are often the only antibiotics that retain activity against MDR, NDM-producing K. pneumoniae strains, they are used as a last-line therapy against these life-threatening superbug infections (8). Alarmingly, polymyxin resistance has started to emerge worldwide, with the most common polymyxin resistance mechanisms in K. pneumoniae involving the activation of the PhoPQ two-component system (TCS), including mutations in *mgrB*, *phoP*, or *phoQ*. Other common resistance mechanisms involve the activation of the PmrAB TCS or the transmission of the plasmid-borne *mcr* genes (26, 27). The activation of the TCSs usually leads to remodeling of the OM, such as the addition of L-ara4N (by the *phoP*/*Q* TCS) or pEtN (by the *pmrA*/*B* TCS or MCR-1) to C-1-/C-4′-phosphate on lipid A.

In this study, we employed lipid A profiling, genomics, OM lipidomics, and metabolomics to elucidate the mechanism(s) of polymyxin resistance in the polymyxin-susceptible, MDR, NDM-producing K. pneumoniae clinical strain S01 and its paired polymyxin-resistant mutant S01R, which contains an in-frame deletion in PhoQ (V258del). The homolog of PhoQ has been well studied in Escherichia coli (19). Amino acid alignment revealed the deletion of V258 of PhoQ in S01R and located this deletion in the HAMP domain, an area where deletions may destroy the central signal converters in bacterial chemotaxis receptors and chemosensory histidine kinases, affecting PhoQ’s autokinase activity, phosphate transfer, and phosphatase ability (19, 28, 29). Lipid A profiling revealed that the addition of L-Ara4N to C-1- and/or C-4′-phosphate of lipid A occurred in S01R (Fig. 1). Metabolomics also confirmed a dramatic accumulation of UDP-L-Ara4FN, an important precursor of L-Ara4N (Fig. 3) (24). These results demonstrated that PhoQ^V258del^ led to the activation of the *arn* operon. Surprisingly, there was also a marginal level of L-Ara4N-modified lipid A in S01 in the absence of polymyxin exposure. Lipid A modification by hydroxylation (-OH) (which is regulated by *lpxO*) (30) was observed in S01 prior to polymyxin B treatment (Fig. 1). These rare modifications of lipid A in S01 conferred a moderate degree of polymyxin resistance (MIC of 2 mg/L) compared to the polymyxin resistance of other K. pneumoniae strains (MICs of <0.5 mg/L) (31). Although there is demonstratable proof that PhoP directly regulates *lpxO*, the latter is often coactivated in the presence of mutations in *phoP* or *phoQ* (32), and this would explain the hydroxylation of lipid A in the mutant strain (Fig. 1). Collectively, the lipid A profiling results showed that a small proportion of lipid A in S01 was modified either by hydroxylation or L-Ara4N, whereas a large proportion of lipid A in S01R permanently contained these modifications. This would explain the extremely high polymyxin B MIC (>128 mg/L) of S01R. Notably, other modifications previously reported to confer polymyxin resistance in K. pneumoniae, such as palmitoylation or the addition of phosphoethanolamine, were not detected in either strain in this study (13, 31).

Previous studies showed a significant association between bacterial metabolic changes and polymyxin resistance in Pseudomonas aeruginosa and Acinetobacter baumannii (33, 34). In these studies, the same polymyxin B concentration (i.e., 4 mg/L) was employed to treat the susceptible P. aeruginosa strain (MIC of 1 mg/L) and its paired resistant strain (MIC of 16 mg/L). The findings suggest that the use of a low polymyxin concentration to stimulate a response from a polymyxin-resistant strain may be insufficient to observe any dramatic metabolome perturbations (31, 33). In this respect, it is noteworthy that due to the difficulty of treating lung infections, inhaled polymyxins have been used in the clinic to achieve significantly higher drug exposures (e.g., >100 mg/L) (35). Therefore, to reflect this clinically relevant scenario, in the present study, we investigated the metabolism changes under a higher concentration of polymyxin B for strain S01R.

The lipopolysaccharide-containing OM of Gram-negative bacteria provides protection from environmental harm (36). Membranes are formed by amphiphilic lipids, which in most cases and conditions studied are GPLs, composed of a glycerol moiety, two fatty acids, a phosphate group, and a variable head group. Examples are PE, PG, CL, lysoPG, PI, PA, and phosphatidylserine (PS) (36). Usually, the outer leaflet of the OM is formed by lipid A, the lipophilic anchor of lipopolysaccharide (LPS), as in Gram-negative bacteria like K. pneumoniae (37). PE and PG are the two major components of OM, while others, such as PC and PI, contribute less than 5% but play a role in bacterial functions, such as virulence (38). Lipid A modification is shown to play a major role in polymyxin resistance in previous work (31, 33), and our present data also highlight this phenomenon (Fig. 1). Apart from lipid A, the GPLs in the bacterial OM also play an important role in polymyxin activity, as polymyxin can discriminately bind to PE and PG (34). The OM lipidomics data showed differential regulation in GPLs and fatty acids in S01R compared to their regulation in S01, with an increase of PE possibly contributing to increased polymyxin resistance. For the synthesis of those components, lysoPE and lysoPG are the intermediates to PE and PG. After polymyxin treatment, accumulation of lysoPE and lysoPG was detected by liquid chromatography-mass spectrometry (LC-MS) in both S01 and S01R, showing dysfunction in the metabolism of these GPLs. However, there was greatly increased fatty acid and GPL biosynthesis following polymyxin exposure in both strains (Fig. 5). Acyl-CoA is also an essential substrate of GPL synthesis, and the induced lipid synthesis might act as a sink of intracellular acyl-CoA. From the viewpoint of metabolism changes, treating the resistant strain with a very high concentration of polymyxin (>100 mg/L) also elicits a significant metabolic change that is similar to what occurs in the susceptible strain at a lower level of drug exposure (4 mg/L). Therefore, even without taking the polymyxin-induced nephrotoxicity into account (39), purely increasing the polymyxin concentration is not effective in combating polymyxin-resistant strains; combination with other antibiotics, such as chloramphenicol (22), or even bacterial phage therapy (40) could be better options.

### Conclusions.

This study reveals that a new mutation, V257del, in PhoQ leads to high-level polymyxin resistance along with an L-Ara4N modification to lipid A. Paired metabolomics of K. pneumoniae strains S01 and S01R treated with polymyxin B has shown membrane remodeling of strain S01R that makes it more resistant to polymyxin B. Bacterial cells of strains S01 and S01R reduced their energy and amino acid elements and raised their levels of lipids and fatty acids to counter the insult of polymyxin B. Overall, our findings highlight that MDR, NDM-producing K. pneumoniae can rapidly attain high-level polymyxin resistance through metabolome plasticity that markedly ramps up the production of intermediates that feed into the aminoarabinose lipid A modification pathway.

## MATERIALS AND METHODS

### Antibiotic, bacterial strains, media, and MIC testing.

Polymyxin B sulfate (PMB; lot no. 20120204) was obtained from Beta Pharma (Zhejiang, China). Prior to each experiment, sterile stock solutions of polymyxin B were prepared using Milli-Q water (Millipore, USA) and filtration through a 0.22-μm syringe filter (Sartorius, Germany). A previously characterized polymyxin-susceptible uropathogenic isolate of K. pneumoniae, strain S01, obtained from the urine of a Thai patient (20), was used as the initial bacterial strain. To screen for polymyxin-resistant mutants, 100 μL of appropriately diluted S01 bacterial log-phase culture (cation-adjusted Mueller-Hinton broth [CAMHB]; Oxoid, UK) was plated on Mueller-Hinton agar containing 10 mg/L polymyxin B. The polymyxin B MICs of S01 (2 mg/L) and the polymyxin-resistant mutant S01R (>128 mg/L) were determined by broth microdilution in triplicate on three separate days according to Clinical and Laboratory Standards Institute (CLSI) guidelines (41).

Prior to each study, S01 was cultured on antibiotic-free Mueller-Hinton agar (MHA) and its polymyxin-resistant mutant S01R on MHA containing 10 mg/L polymyxin B. A single colony of each strain was then randomly selected and grown overnight in CAMHB for 16 to 18 h. Subsequently, 1 mL of the overnight culture was diluted 100-fold in prewarmed CAMHB and further grown until mid-log-phase (optical density at 600 nm [OD_600_] of 0.5 ± 0.02). All liquid cultures were incubated at 37°C with shaking (180 rpm).

### *In vitro* passaging.

Log-phase cultures (OD_600_ of 0.5, approximately 10^8^ CFU/mL) of K. pneumoniae S01 and S01R were inoculated (1:10 vol/vol dilution) into 200 μL fresh CAMHB containing no polymyxin B in a 96-well microplate with 4 biological replicates. The cultures were incubated at 37°C for 12 h before the next dilution (1:10, vol/vol) with fresh CAMHB. Successive subculturing was conducted for 7 days.

### DNA sequencing and genomic analysis.

Genomic DNA of S01R was extracted from 2 mL of overnight bacterial culture using a DNeasy blood and tissue kit (Qiagen, Germany) following the manufacturer’s instructions. Genomic DNA quality and quantity were checked using electrophoresis and the NanoDrop 1000 instrument (Thermo Fisher Scientific). Single-ended, 75-bp DNA sequencing was performed on an Illumina MiSeq next-generation sequencer (Genewiz, China). DNA-Seq raw reads were trimmed by Nesoni-clip and aligned to the S01 reference genome using Subread (42). Single nucleotide variations were detected by freebayes (43), filtered by Nesoni vcf-filter (44), and annotated by SnpEff (45). Structural variations were detected by GRIDSS (46) using default settings.

### Time-kill study.

Static time-kill studies were used to examine bacterial killing in the absence (growth controls) and presence of polymyxin B against K. pneumoniae strain S01 and its mutant S01R. We used two conditions. The typical study started with an inoculum of 10^6^ CFU/mL for a standard time-kill study. We also measured the time-kill with an inoculum of 10^8^ CFU/mL, which was the inoculum used for the metabolomics study. Polymyxin B for S01 was investigated at 0, 2, and 4 mg/L under both inoculum conditions. At the 10^6^-CFU/mL inoculum, polymyxin B for S01R was investigated at 0, 128, and 256 mg/L, while at the 10^8^-CFU/mL inoculum, it was investigated at 0, 256, and 512 mg/L. Viable-cell counting was conducted at 0, 1, 4, and 24 h.

### Sample preparation of lipid A.

Lipid A was isolated by the mild acid hydrolysis method as previously described (33). Briefly, ~100 mL of 10^8^-CFU/mL cell cultures of S01 and S01R were harvested. The collected bacterial cells were lysed with a single-phase Bligh-Dyer mixture (chloroform/methanol/water, 1:2:0.8 [vol/vol]) and then resuspended in 10.8 mL of hydrolysis buffer (50 mM sodium acetate, pH 4.5) before incubating in a boiling water bath for 45 min. After cooling to room temperature, 12 mL chloroform and 12 mL methanol were added to the 10.8-mL hydrolysis solution containing the cell sample to make a double-phase Bligh-Dyer mixture (chloroform/methanol/water, 1:1:0.9 [vol/vol]). The lower phase containing lipid A was collected and air-dried under a fume hood for future use.

### Sample preparation of outer membrane lipids.

Samples for membrane lipidomics were prepared as described previously (47). Briefly, 400-mL amounts of bacterial cells at ~2 × 10^8^ CFU/mL from strains S01 and S01R were pelleted separately and resuspended in 5 mL of 0.75 mM sucrose in 10 mM Tris-HCl, pH 7.5, and then 2.5 μL lysozyme (50 μg/mL), 200 μL phenylmethylsulfonyl fluoride (2 mM), and 10 mL 1.65 mM EDTA, pH 7.5, were added. Cell homogenization was then performed using EmulsiFlex (Avestin, Canada) at 15,000 lb/in^2^. Total membranes were collected by ultracentrifugation (38,000 rpm for 45 min at 4°C; Beckman Coulter, USA) and resuspended in 25% sucrose (wt/vol) in 5 mM EDTA. The inner and outer membranes were separated by a six-step sucrose gradient (35, 40, 45, 50, 55, and 60% sucrose [wt/vol] in 5 mM EDTA, pH 7.5) through ultracentrifugation with an SW40 Ti rotor (34,000 rpm for 17 h at 4°C). Membrane fractions (1 mL) were collected using a fraction collector (Teledyne ISCO, USA) with 70% sucrose (wt/vol) in 5 mM EDTA, pH 7.5, as the displacement fluid. Experiments were performed in duplicate, and all samples stored at −80°C prior to analysis.

### Sample preparation of metabolites.

Polymyxin B concentrations of 4 mg/L for S01 and 512 mg/L for S01R were chosen based on preliminary time-kill studies (inoculum of ~8.5 log_10_ CFU/mL) that showed minimal bacterial killing (~3 and 0.5 log_10_ CFU/mL, respectively) at these concentrations. Intracellular metabolites were extracted as described previously (33). First, 10 mL of ~10^8^ CFU/mL bacterial cells were collected at 1 h and 4 h for each sample, with four replicates. Second, the harvested cultures were quenched in a dry ice/ethanol bath. Cellular metabolites were extracted using 250 μL of a chloroform/methanol/water mixture (1:3:1 [vol/vol]) containing internal standards (CHAPS {3-[(3-cholamidopropyl)-dimethylammonio]-1-propanesulfonate}, CAPS (*N*-cyclohexyl-3-aminopropanesulfonic acid), PIPES [piperazine-*N*,*N*′-bis(2-ethanesulfonic acid)], and Tris, all at 1 μM) with centrifugation (14,000 × *g* for 10 min at 4°C). Finally, 200 μL of each sample was transferred to an injector vial for LC-MS analysis (described below).

### LC-MS analysis for lipid A, lipids, and metabolites.

Measurement of lipid A profiles, membrane lipids, and whole-cell metabolites was conducted on a Dionex U3000 high-performance liquid chromatography (HPLC) system in tandem with a Q-Exactive Orbitrap mass spectrometer (Thermo Fisher Scientific) as previously described (33). The electrospray voltage was set at 3.50 kV, and nitrogen was used as the collision gas. All samples were randomized in each of the omics studies.

Structural and semiquantitative analyses of lipid A were conducted in negative mode with resolution at 70,000 and a mass range from 167 to 2,500 *m/z*. The Synergi Hydro-RP 80-Å column (50 by 2 mm, 4-μm particle size; Phenomenex, USA) was maintained at 40°C. Lipid A samples maintained at 4°C were eluted with mobile phase A (40% isopropanol and 60% water containing 8 mM ammonium formate and 2 mM formic acid) and mobile phase B (98% isopropanol and 2% water containing 8 mM ammonium formate and 2 mM formic acid). The flow rate was 0.2 mL/min for the first 16 min and was increased to 0.5 mL/min from 16 to 22 min. The elution started with 70% mobile phase A and 30% mobile phase B, followed by a linear gradient to a final composition of 100% mobile phase B, which was maintained for 4 min. Lipidomics analysis was conducted in both positive and negative modes with resolution at 35,000. The Ascentis Express C_8_ analytical column (product number 53831-U, 5 cm by 2.1 mm, 2.7-μm particle size; Sigma-Aldrich) was maintained at 40°C, and the samples were kept at 4°C. The flow rate was 0.2 mL/min for the first 25 min and was increased to 0.5 mL/min from 25 to 30 min. The linear gradient started from 100% mobile phase A and was reduced to a final composition of 35% mobile phase A and 65% mobile phase B. Metabolomics samples were analyzed by a full MS scan in both positive and negative modes at a resolution of 35,000 with a mass range from 85 to 1,275 *m/z*. A ZIC-pHILIC column (SeQuant, 5-μm particle size, polymeric, 150 by 4.6 mm; Merck) was maintained at 25°C. The LC elution buffer consisted of 20 mM ammonium carbonate (A) and acetonitrile (B) with a multistep gradient system going from 80% B to 50% B over 15 min and then reducing to 5% B at 18 min, followed by washing with 5% B for 3 min; the flow rate was 0.3 mL/min. The chromatographic peaks, signal reproducibility, and analyte stability were monitored by assessment of pooled biological quality control (PBQC) samples (with a 10-μL aliquot of each sample) analyzed periodically throughout the batch, internal standards, and total ion chromatograms for each sample. Mixtures of pure standards containing over 300 metabolites were analyzed within the batch to aid metabolite identification.

### Data processing.

Untargeted lipidomic and metabolic analyses were performed through mzMatch (48) and IDEOM (http://mzmatch.sourceforge.net/ideom.php) (49). The proteoWizard tool msconvert was used to convert raw LC-MS data files to the mzXML format. Automated chromatography peaks were picked by XCMS (50), converted to PeakML files, and combined and filtered on the basis of the intensity (1,000), reproducibility (relative standard deviation [RSD] for all replicates, <0.8), and peak shape (codadw >0.8) by mzMatch. Missing peaks were retrieved and related peaks annotated by the mzMatch program. Unmatched peaks and noises were rejected through IDEOM. Putative metabolites were identified via the neutral exact mass within 3 ppm and confirmed by the retention times of authentic standards (<5%) and the calculated retention times (<50%). The databases used in IDEOM included KEGG (51), MetaCyc (52), and LIPID MAPS (53). Principal-component analysis (PCA) data were plotted in MetaCyc. Peak intensity was used to quantify each metabolite, and the values compared with those of the wild-type and mutant strains. Univariate statistical analysis was performed using both one-way analysis of variance (ANOVA) and Student’s *t* test (*P* < 0.05).

### Data availability.

The complete genome of Klebsiella pneumoniae strain S01 has been sequenced and deposited in GenBank with accession number PRJNA604394. The data sets used and/or analyzed during the current study are available from the corresponding author on reasonable request.
