# Supplementary material for: Lipid A Modification and Metabolic Adaptation in Polymyxin-Resistant, New Delhi Metallo-β-Lactamase–Producing Klebsiella pneumoniae
Source: Microbiol Spectr. 2023 Jul 11;11(4):e00852-23. doi: 10.1128/spectrum.00852-23 (PMC10433984; doi:10.1128/spectrum.00852-23)
Supplement: Supplemental file 3 — Figure S1-5 and Table S1.. Download spectrum.00852-23-s0003.docx, DOCX file, 4.1 MB [file spectrum.00852-23-s0003.docx]

**Supplementary Information for**

**Lipid A modification and metabolic adaptation in polymyxin-resistant New Delhi metallo-β-lactamase‑producing *Klebsiella pneumoniae***

Jing Lu^1,#^, Meiling Han^1,#^, Heidi H. Yu^1^, Phillip J. Bergen^1^, Yiyun Liu^2^, Jinxin Zhao^1^, Hasini Wickremasinghe^1^, Xukai Jiang^1,3^, Yang Hu^1^, Haiyan Du^1,4^, Yan Zhu^1,5,*^, Tony Velkov^6,*^

*Correspondence to: Associate Professor Tony Velkov, Email: [Tony.Velkov@monash.edu](file:///G:\Shared%20drives\Seq%20Analysis_Jing%20and%20Yan\Kp02\Tony.Velkov@monash.edu). and Dr Yan Zhu, Email: [Yan.Zhu@monash.edu](mailto:Yan.Zhu@monash.edu).

#Equal contribution.

**This file includes:**

Supplementary figures

**Supplementary Figures 1 to 5**

**Supplementary table**

**Supplementary Table 1**

**Titles for Supplemental Data files**

**Data S1**

**Data S2**

Supplementary figures

Supplementary Figure S1


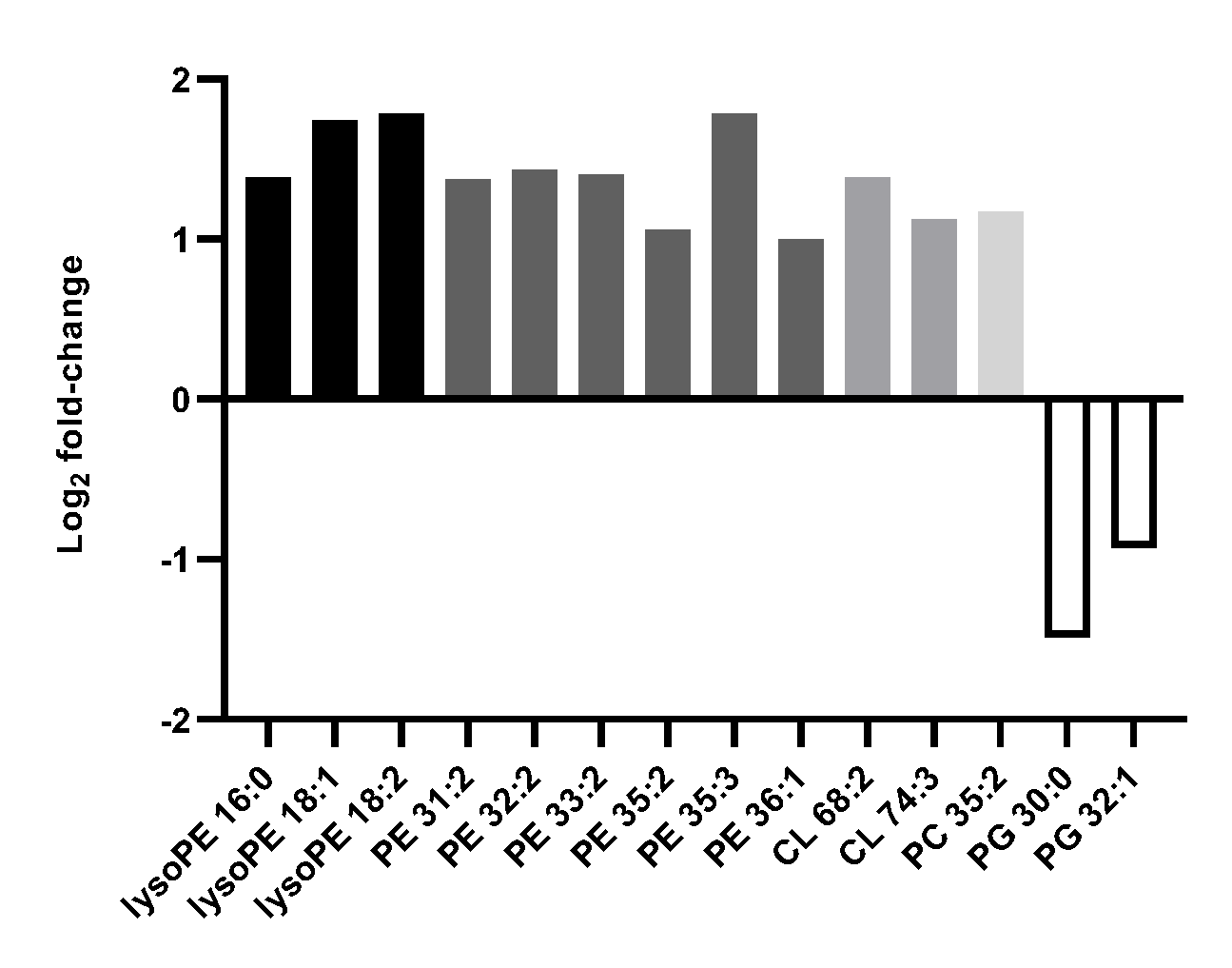


**Figure S1. Lipid composition and significantly changed lipids in the outer membrane of S01R compared to S01.** The significantly changed glycerophospholipids in S01R compared to S01. Lipid composition was determined following 4 h of growth in polymyxin-free, cation-adjusted Mueller Hinton broth. lysoPE, lysophosphatidylethanolamine; PE, phatidylethanolamine; CL, cardiolipin; PC, phosphatidylcholine; PG, phosphatidylglycerol.

Supplementary Figure S2

A


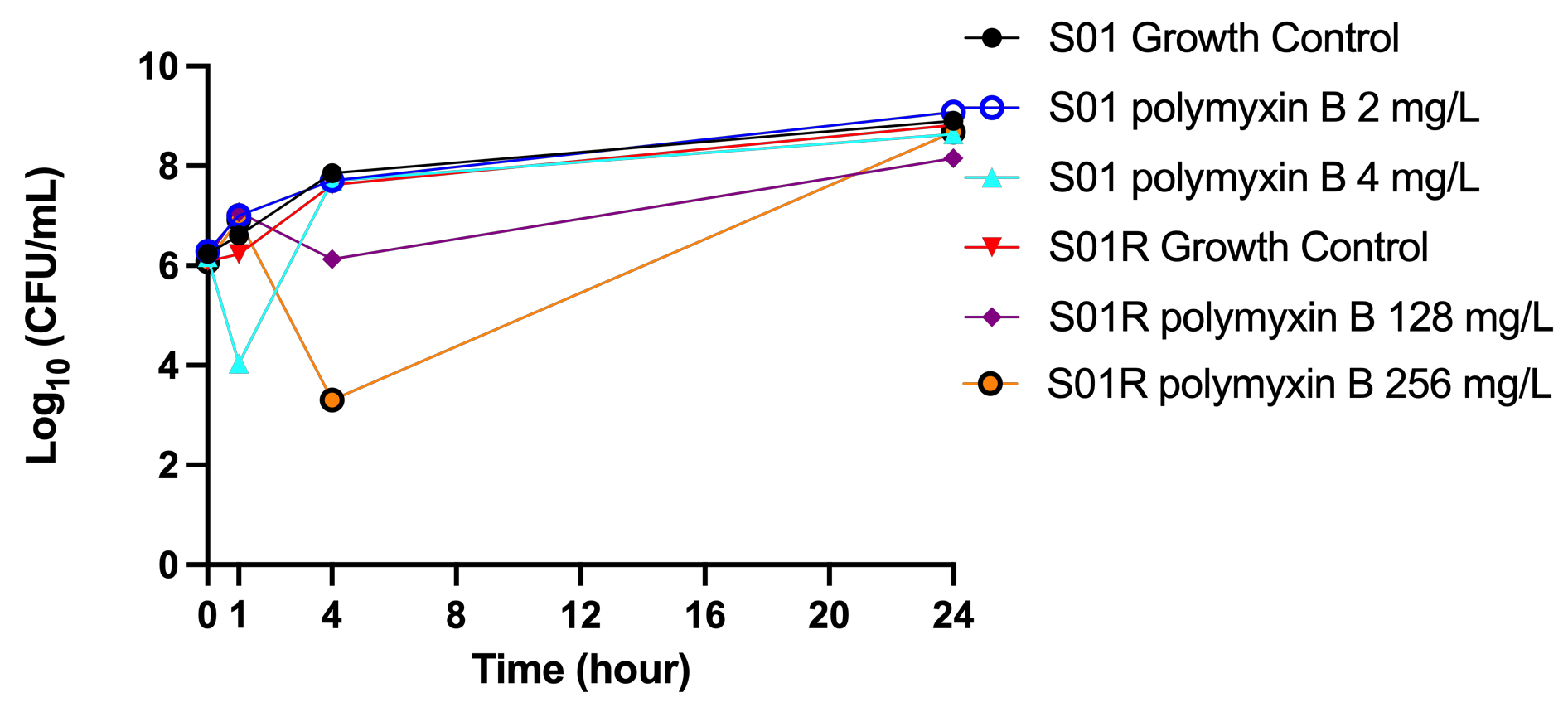


B


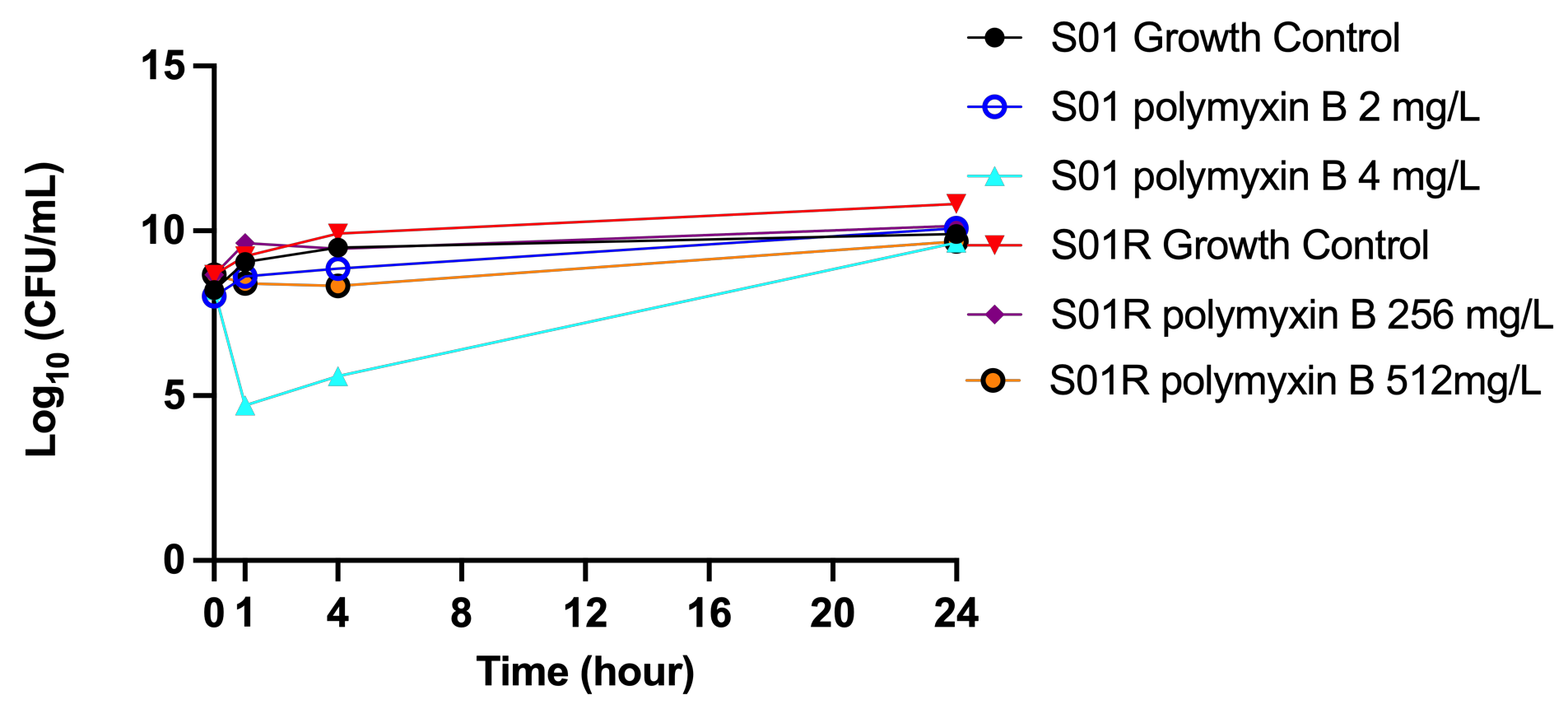


Figure S2. Time–kill curves of Kp S01 and S01R with various concentrations of polymyxin B with an inoculum of 10^6^ (A) or 10^8^ (B) CFU/mL.

**Supplementary Figure S3**

**
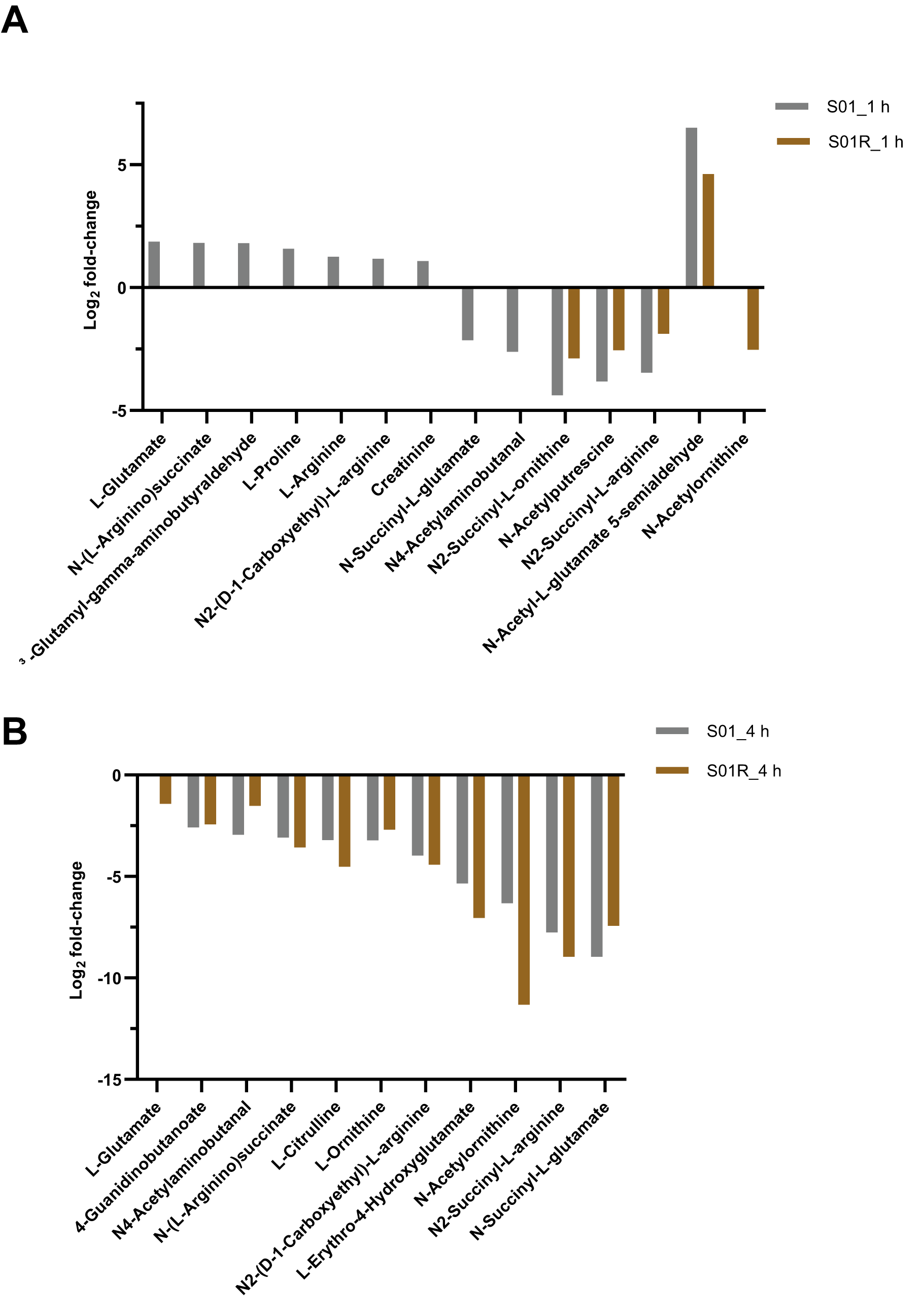
**

**Figure S3. Changes in arginine and proline following polymyxin treatment.** (**A**, **B**) Log_2_ fold-changes of significantly changed metabolites involved in arginine and proline metabolism following 1 h (**A**) and 4 h (**B**) of polymyxin B exposure.

**Supplementary Figure S4**

**
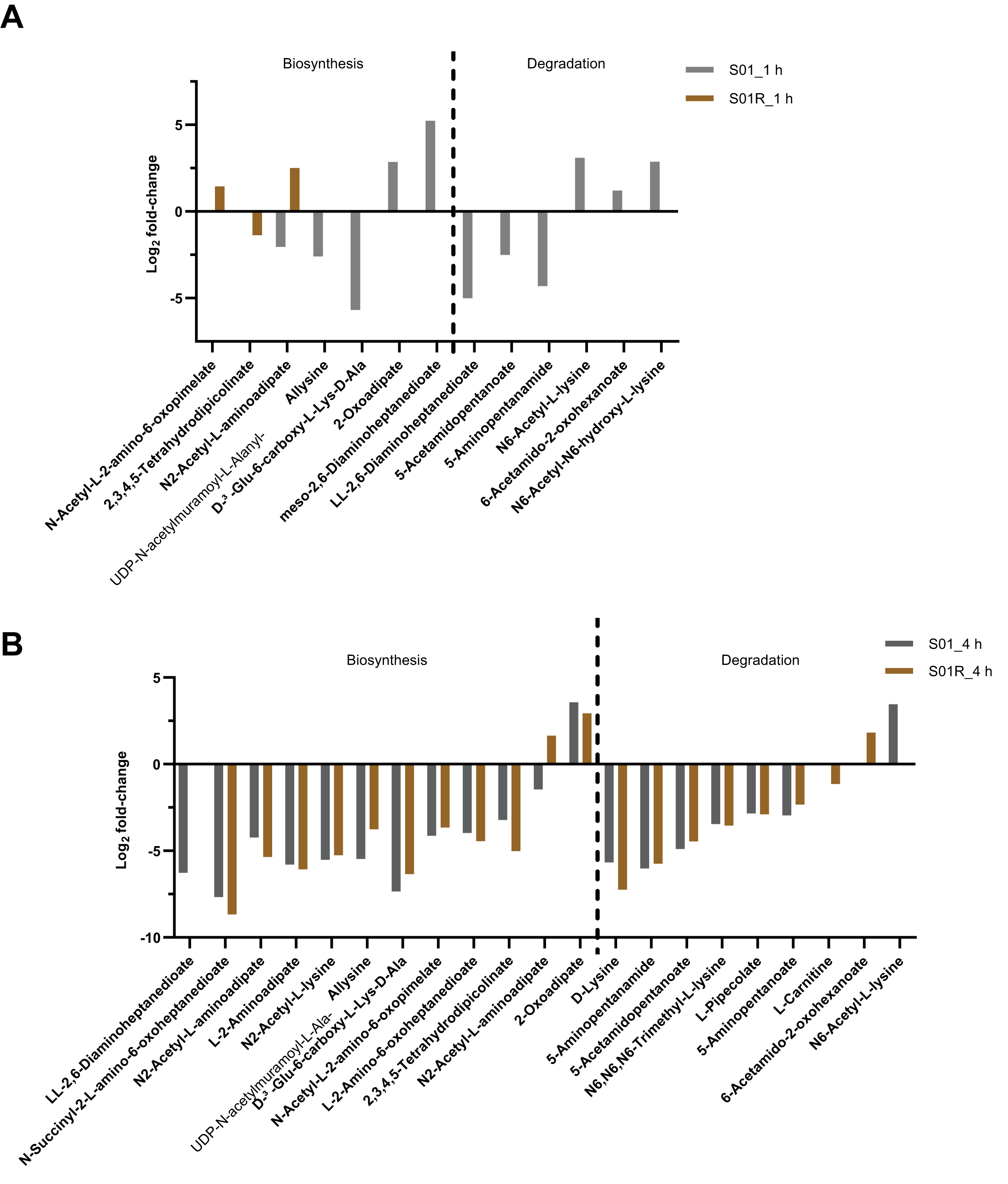
**

**Figure S4. Changes in lysine metabolism following polymyxin treatment. (A, B**) Log_2_ fold-changes of significantly changed metabolites involved in lysine biosynthesis and degradation following 1 h (**A**) and 4 h (**B**) of polymyxin B exposure.

**Supplementary Figure S5**

**
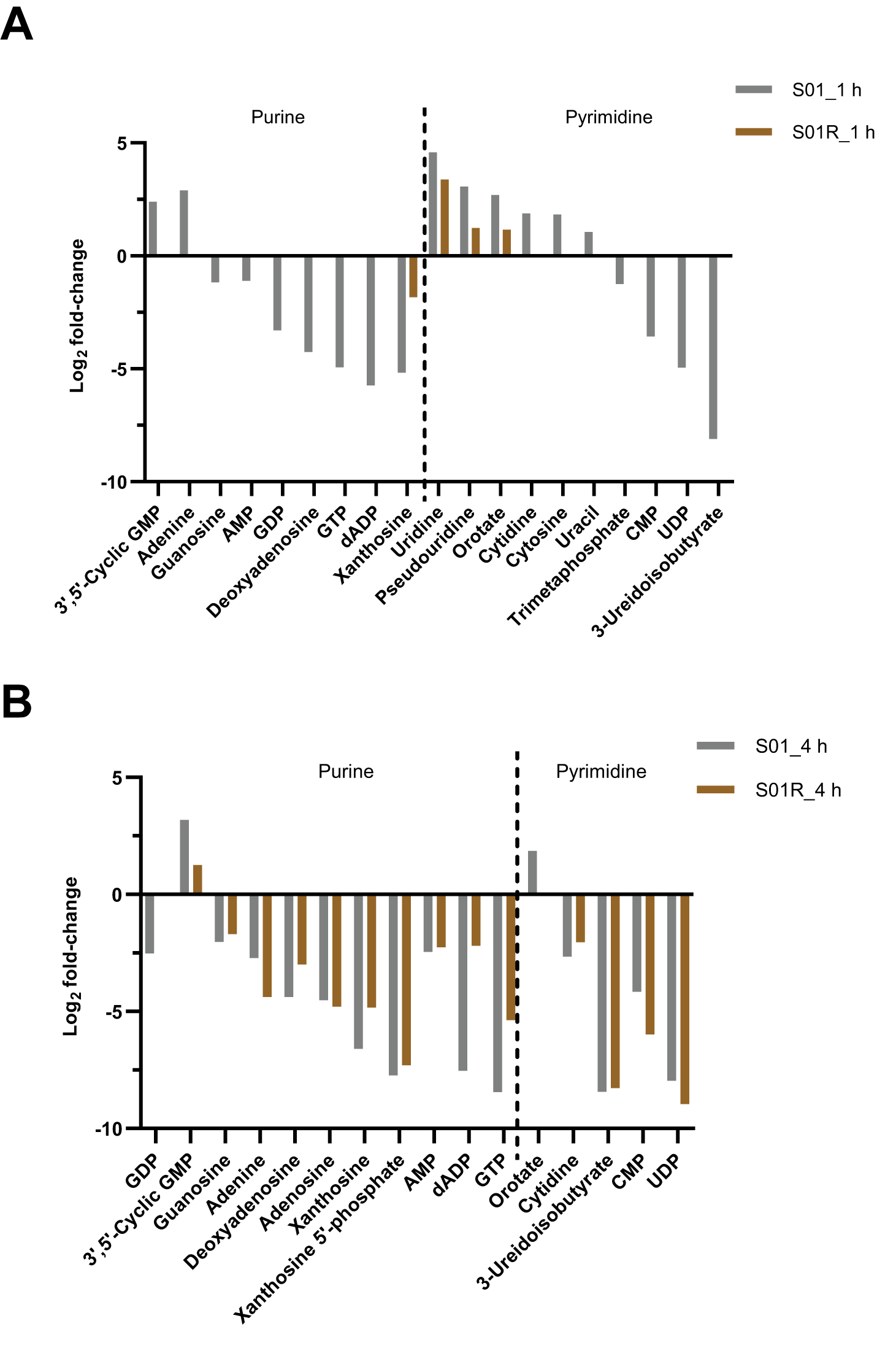
**

**Figure S5. Changes in purine and pyrimidine metabolism following polymyxin treatment.** (**A, B**) Log_2_ fold-changes of significantly changed metabolites involved in purine and pyrimidine metabolism following 1 h (**A**) and 4 h (**B**) of polymyxin B exposure.

**Supplementary Table**

|  | Replicate | Day 1 | Day 2 | Day 3 | Day 4 | Day 5 | Day 6 | Day 7 |
| --- | --- | --- | --- | --- | --- | --- | --- | --- |
| Kp  S01 | R1 | 2 | 2 | 2 | 2 | 2 | 2 | 2 |
|  | R2 | 2 | 2 | 2 | 2 | 2 | 2 | 2 |
|  | R3 | 2 | 2 | 2 | 2 | 2 | 2 | 2 |
|  | R4 | 2 | 2 | 2 | 2 | 2 | 2 | 2 |
| Kp S01R | R1 | 256 | 256 | 256 | 256 | 256 | 256 | 256 |
|  | R2 | 256 | 256 | 256 | 256 | 256 | 256 | 256 |
|  | R3 | 256 | 256 | 256 | 256 | 256 | 256 | 256 |
|  | R4 | 256 | 256 | 256 | 256 | 256 | 256 | 256 |

**Supplementary Table 1. Stability of polymyxin B resistance during *in vitro* passaging of *K. pneumoniae* S01 and S01R every 24 hour in CaMHB without polymyxin B by measuring the minimum inhibitory concentration (MICs) (*n* = 4).**

**Titles for Supplemental Data files**

**Data S1. The significant changed metabolites in phospholipids in S01 and S01R following polymyxin B treatment.**

**Data S2. The significant changed metabolites in fatty acids in S01 and S01R following polymyxin B treatment.**
